# Supplementary material for: The Construction and Exploration of a Comprehensive MicroRNA Centered Regulatory Network in Foxtail Millet (Setaria italica L.)
Source: Front Plant Sci. 2022 May 6;13:848474. doi: 10.3389/fpls.2022.848474 (PMC9121102; doi:10.3389/fpls.2022.848474)
Supplement: Supplementary file 2 [file Data_Sheet_2.zip › Sit-miR5568_Seita.9G150400.1_380_TPlot.pdf]

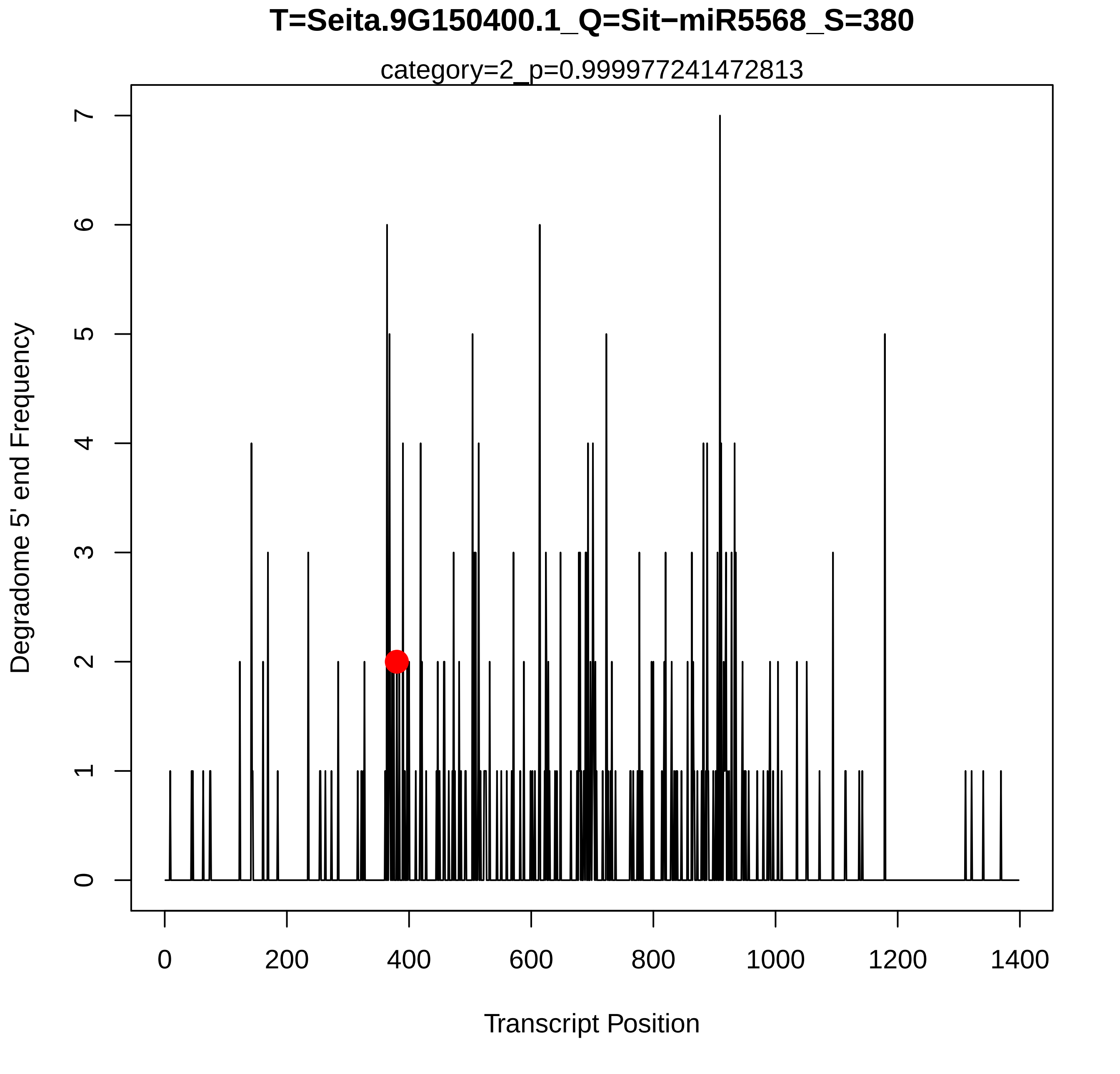
T=Seita.9G150400.1_Q=Sit−miR5568_S=380
category=2_p=0.999977241472813
Degradome 5' end Frequency
0
1
2
3
4
5
6
7
Transcript Position
0
200
400
600
800
1000
1200
1400
